# Molecular Detection and Characterization of *Theileria* Infecting Wildebeest (*Connochaetes taurinus*) in the Maasai Mara National Reserve, Kenya

**DOI:** 10.3390/pathogens4030626

**Published:** 2015-08-18

**Authors:** Lucy Wamuyu, Vincent Obanda, Daniel Kariuki, Francis Gakuya, Moni Makanda, Moses Otiende, Sheila Ommeh

**Affiliations:** 1 Department of Biochemistry, Jomo Kenyatta University of Agriculture and Technology (JKUAT), P.O. Box 62000-00200, City Square, Nairobi, Kenya; E-Mails: wamuyulucy27@gmail.com (L.W.); dkariuki@jkuat.ac.ke (D.K.); 2 Department of Veterinary Services, Kenya Wildlife Service, P.O. Box 40241-00100, Nairobi, Kenya; E-Mails: gakuya@kws.go.ke (F.G.); motiende@kws.go.ke (M.O.); 3 Institute For Biotechnology Research (IBR), Jomo Kenyatta University of Agriculture and Technology (JKUAT), P.O. Box 62000, City Square 00200, Nairobi, Kenya; E-Mails: monimakanda9@gmail.com (M.M.); sommeh@jkuat.ac.ke (S.O.)

**Keywords:** wildlife-livestock interface, tick-borne diseases, ticks, *Theileria*, wildlife

## Abstract

*Theileria* is a genus of tick-borne protozoan that is globally widespread and infects nearly all ungulates in which they cause either latent infection or lethal disease. Wild animals are considered reservoir hosts of many species of *Theileria* and their diversity in wildlife species is increasingly becoming of interest. The molecular characterization and identification of *Theileria* infecting wildlife has been studied in a few species including buffalo, which are considered reservoir host for *Theileria parva* infecting cattle. In this study, we sequenced *Theileria* species infecting wildebeest (*Connochaetes taurinus*) and used molecular-genetic and phylogenetic analysis of the 18 Small Subunit of the Ribosomal RNA (18S rRNA) to identify their relationships with known species of *Theileria*. Our results revealed three new *Theileria* haplotypes infecting wildebeest. Phylogenetic analysis revealed that haplotype 1 and 2 clustered in the same clade as *Theileria separata* and with *Theileria* sp. isolated from other small to medium sized antelopes. Haplotype 3 clustered close to the *Theileria ovis* clade. This is the first molecular description and characterization of *Theileria* species infecting blue wildebeest in East Africa. This study demonstrates the potential for *Theileria* transmission between wildebeest and small domestic ungulates, such as sheep and goats.

## 1. Introduction

*Theileria* are protozoan parasites that infect a wide range of wild and domestic ungulates worldwide, causing diseases that are of great economic importance because of their impact on livestock health and production [1,2,3]. In Africa, the economic costs of Theileriosis are estimated at US $168 million from cattle mortality alone [3]. *Theileria* species are transmitted among hosts by several species of Ixodid tick vectors [4]. Many wildlife species harbor a great diversity of *Theileria* species and infected individuals are usually asymptomatic carriers [5,6,7]. The high prevalence of and the prolonged carrier state of *Theileria* infections in many wildlife populations studied so far suggests that wildlife species are reservoir hosts for *Theileria* species infecting domestic ungulates [5,6,7,8,9]. The role of wildlife as reservoir hosts (because they have high prevalence and asymptomatic infections of *Theileria* species) has stimulated research on the molecular characterization, identification and epidemiology of *Theileria* infecting wildlife. Most of these studies however, have focused on the role of buffalo as a reservoir for *Theileria parva lawrenci*, which causes lethal corridor disease in cattle. More recently, studies on wildlife *Theileria* has expanded to include a number of wildlife host species, such as grants’ gazelle (*Nanger granti*) [10], waterbuck (*Kobus ellipsiprymnus*) [11], giraffe (*Giraffa camelopardis*) [12] zebras (*Equus quagga*) [13,14], and black rhinoceros (*Diceros bicornis*) [15]. Early microscopic and serological studies in blue wildebeest isolated and identified *Theileria gorgonis* [8]. However, little has since been done regarding *Theileria* species infecting wildebeest, which are particularly important as conduits of livestock diseases because of their annual long distance migrations for example in the Serengeti-Mara ecosystem in East Africa and in the Kalahari [16,17]. Long distance migration is suggested to play significant role in the spread of parasites [18]. For example Morgan *et al*. [19] demonstrated how seasonal movement of Saiga antelope (*Saiga tatarica tatarica*) influenced the spread of helminths within sheep populations along their migratory route [19]. Historically, the migration of wildebeests in East Africa has been associated with the spread of Malignant Catarrhal Fever (MCF), which is a deadly viral disease in cattle [20]. The role of long distance mammalian migration in the transmission of tick borne pathogens has not received much research attention.

Although wildlife harbors a great diversity of parasites, usually in latent form, they still exert deleterious effects on their hosts and influence their host populations directly or indirectly [21,22]. Like many parasitic diseases of wildlife, *Theileria* infections are often latent but can progress to fatal disease under conditions of either nutritional or translocation stress [23,24,25,26,27]. Several cases of mortalities caused by *Theileria* infection have been documented in black rhinoceros and plains zebra as well as several species of antelopes, such as roan, sable, eland, greater kudu, gray duiker and tsessebe [28,29,30]. Infections by *Theileria* have also been implicated as a cause for high calf mortality responsible for the decline in populations of roan and sable antelopes in South Africa [27,31].

The use of polymerase chain reaction (PCR) and sequencing in the diagnosis and detection of protozoans has led to the discovery of unparalleled species diversity in the genus *Theileria* and also in the revelation of new hosts for *Theileria* species that were thought to be host specific [32]. Knowledge of the genetic polymorphism in the 18S rRNA of *Theileria* is essential in designing molecular-genetic probes for the detection of species and strains of *Theileria* of economic importance to livestock production and wildlife conservation [12,33].

Here, we used molecular genetic techniques, involving PCR and sequencing of the 18s rRNA gene to detect and characterize species of *Theileria* infecting migratory and resident wildebeest in the Serengeti-Mara ecosystem.

## 2. Results

**Figure 1:**
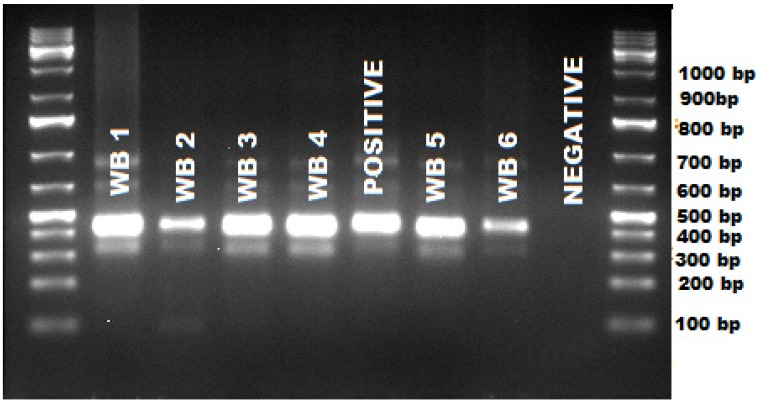
Gel image showing positive amplification of *Theileria* sp. in wildebeest and amplicon sizes in base pairs.

PCR and gel analysis revealed that all the 32 wildebeest blood samples were positive for either *Theileria* or *Babesia* since the primers we used could amplify both species. The amplicon size was found to range between 450–500 bp (Figure 1).

**Figure 2:**
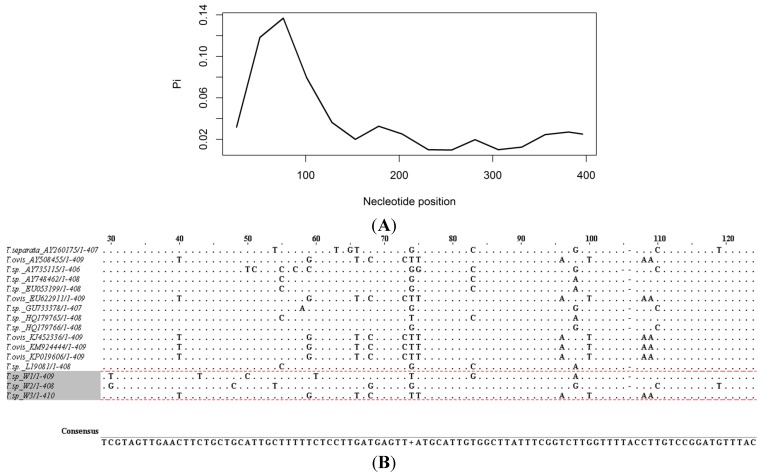
Polymorphism of *Theileria* 18s rRNA gene (**A**) showing that it is localized to a region 1–150 bp from the ILO primers and (**B**) a sequence alignement showing variability in that 1–150 within the three wildebeest haplotypes, *T. ovis* and *T. Seperata*cluster.

We sequenced all the 32 amplicons and obtained 29 good quality sequences while three sequences were of poor quality. The poor quality sequences (3) were discarded from further analysis. Out of the 29 good sequences, 23 were from migratory wildebeest and six were from resident population of wildebeest. We analyzed the sequences and all the 29 good sequences were positive for *Theileria* sp. Sequence analysis revealed three unique *Theileria* haplotypes in wildebeest; *Theileria* sp. wildebeest haplotype 1 (GenBank accession number, KT163244) was identified in one resident and one migratory wildebeest; *Theileria* sp. wildebeest haplotype 2 (GenBank accession number, KT163245) was identified in migratory wildebeest only while *Theileria* sp. wildebeest haplotype 3 (GenBank accession number, KT163246) was identified in five resident wildebeest and 19 migratory wildebeest. Polymorphism in the three haplotypes was elevated in the first 100 bp of the V4 region of 18s rRNA, a property shared by other species (Figure 2).

**Figure 3:**
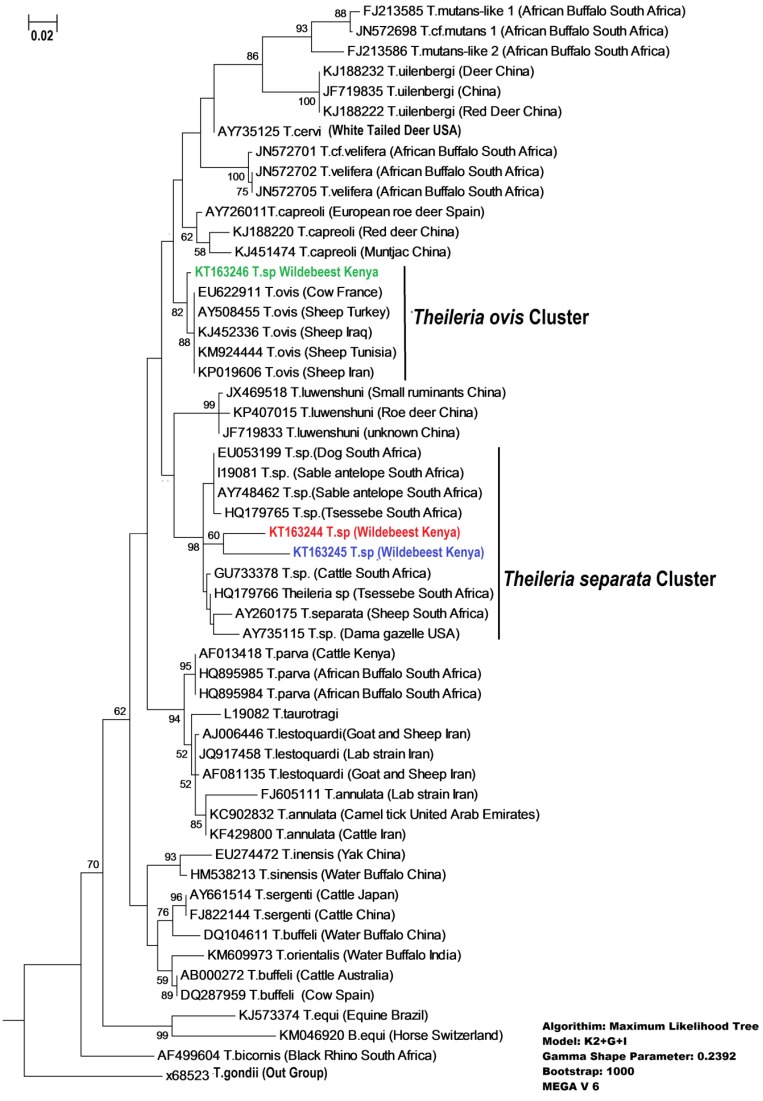
Phylogenetic relationship between *Theileria* isolated from Wildebeest and *Theileria* isolates from the GeneBank based on a 450 bp sequence of the V4 region of 18s rRNA gene. Phylogeny was established using maximum likelihood. Numbers above the branches indicate bootstrap values based on 1000 replicates. *Theileria* isolates from wildebeest (in color) while the rest were accessed from the GeneBank (in black). The gamma shape parameter value was 0.2392 indicating high rate of heterogeneity among the species.

Phylogenetic analysis of our haplotypes with similar sequences based on BLASTn (NCBI) and with sequences of known identity showed a clustering of Haplotype 1 and Haplotype 2 with *Theileria separata* while Haplotype 3 clustered with *Theileria ovis* (Figure 3).

**Figure 4:**
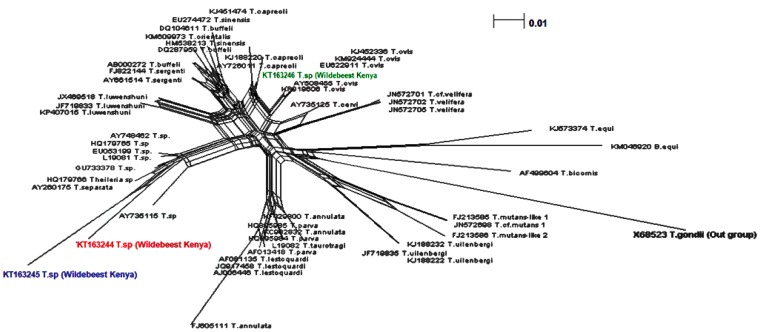
Network graph showing the separation of *Theileria* haplotypes (in color) from wildebeest and isolates from the GeneBank (in black). (Model Fit: 96.234, Taxa: 54, Chars: 462, Algorithim: Uncorrected_P NeighborNet with equal angle).

The phylogenetic network analysis confirmed that our haplotype clustering was consistent with the topology of the phylogenetic tree (Figure 4).

Genetic divergence between *Theileria* haplotypes detected in wildebeest and *Theileria* species they clustered with was variable. There was very limited divergence, especially between *Theileria ovis* and haplotype 3, but greater divergence and heterogeneity between *Theileria separata* with haplotypes 1 and 2 (Table 1).

**Table 1:** Sequence divergence between *Theileria* haplotypes infecting wildebeest and two of the most closely associated sequences of known species obtained from GeneBank.

	Hap 1	Hap 2	Hap 3	*Theileria ovis*	*Theileria separata*
Hap 1		0.075	0.075	0.077	0.059
Hap 2	0.075		0.097	0.100	0.059
Hap 3	0.075	0.097		0.007	0.053
*Theileria ovis*	0.077	0.100	0.007		0.056
*Theileria separata*	0.059	0.05	0.053	0.056	

## 3. Discussion

Our results showed that all the wildebeests were infected with *Theileria*, which is consistent with infection levels in other wild mammalian hosts. For example, Hawkins *et al*. [14] found prevalence of *Theileria equi* in Grevy’s zebra to be 100%. Similarly high levels of infection have been reported in African buffalo populations in Uganda [34].

In this study, we present the first genetic identification of *Theileria* in the East African blue wildebeests. By using advanced molecular techniques we identified three new haplotypes in the wildebeest population in which the most abundant haplotype clustered in *Theileria ovis* clade and the other two were within the *T. separata* cluster (Figure 3). *T. ovis and T. separata* are mainly identified in small or medium sized wild and domestic ruminants. Elsewhere, both *Theileria ovis* and *Theileria separata*, beside *Theileria lestoquardi*, are the cause of ovine theileriosis in sheep and goats [35,36]. However, the occurrence and species of *Theileria* infecting sheep and goats in Kenya are not known. In wildlife, *Theileria* sp. closely related to *Theileria separata* have been identified in grey duiker (*Sylvicapra grimmia*), common tsessebe (*Damaliscus lunatas*) and sable antelope (Hippotragus niger) [26].

Wildlife is considered important reservoir of up to 77% of the livestock diseases [20]. Since most of the *Theileria* species are multi-host parasites, wildebeest, because of their large populations [37,38] and high *Theileria* prevalence, can therefore pose perennial risks of theileriosis to livestock. Several species of *Theileria* have recently been identified in wild mammalian species in Kenya, of which the majority have been novel species or haplotypes [5,11,14,15]. Increasing concern on zoonotics and zoophilic diseases is driving the interests in understanding the diversity of pathogens harbored by wildlife. This interest, coupled by the availability of robust molecular tools, has resulted in the plethora of studies on the detection of novel *Theileria* species and/or haplotypes.

In the present study, two haplotypes (haplotype 1 and 3), were shared between the resident and migratory wildebeests. This suggests that migration of this massive population is potentially playing a central role in disease spread and in homogenizing the distribution of *Theileria* haplotypes across spatially distinct host populations in the Serengeti-Maasai Mara ecosystem. The population of resident wildebeest is estimated to be 31,000, whereas the migratory population that mostly resides on the Serengeti is estimated to be 1.2 Million [37,38]. Migratory wildebeest is the carrier of the wildebeest-derived Malignant Catarrhal fever, which is a lethal viral disease in cattle, and a major constraint in livestock farming among the Maasai pastoralist communities [39].

Although our haplotypes clustered closely with known *Theileria* species, we could not confidently confirm their species identity except for haplotype 3, which clustered with *Theileria ovis*. This haplotype had limited sequence divergence from *T. ovis*. The *Theileria separata* cluster which contained haplotypes 1 and 2 displayed greater sequence divergence within the cluster suggesting that there is either greater genetic heterogeneity in this species or that the cluster contains distinct species that are closely related to *T. separata*. We could not confirm the relationship between our haplotype 1 and 2 with previously confirmed species of *Theileria* (*Theileria gorgonis*) known to infect blue wildebeest due to the lack of sequence data for this species in GenBank [8]. Support for heterogeneity hypothesis within a cluster comes from studies on genetic variation of *T. parva* infecting buffalo and cattle, which have shown that these two hosts can maintain almost independent variants of *T. parva* even when opportunities for cross transmission in sympatry do occur [40,41].

## 4. Materials and Methods

### 4.1. Ethics

Samples used in this research were collected by Kenya Wildlife Service (KWS) for the surveillance of Malignant Catarrhal Fever in wildebeest as part of routine disease surveillance mandate of the Veterinary Services Department of the Kenya Wildlife service. Scientific and ethical approval was provided by the Scientific Research Committee of Kenya Wildlife Service, Reference number KWS/BRM/5001. The protocols and procedures for animal handling and care used during this study follow the Kenya Wildlife Veterinary guidelines and ethical practice 2006.

### 4.2. The Study Area

Maasai Mara National Reserve (MMNR) is located on the South-Western part of Kenya along the Kenya–Tanzania border between 1°13′ and 1°45′ South and 34°45′ and 35°25′ East. It occupies an area of approximately 1510 square kilometers and hosts a high diversity of wildlife including large mammals such as the African elephant (*Loxodonta africana*), lion (*Panthera leo*), leopard (*Panthera pardus*), African buffalo (*Syncerus caffer*), black rhinoceros (*Diceros bicornis*), wildebeests (*Connochaetes taurinus*), and several antelope species [42]. The reserve is contiguous with the Serengeti National Park on the Tanzanian side forming a single ecosystem, the Serengeti-Mara Ecosystem, which is an important habitat for the blue wildebeest migration in East Africa. The MMNR maintains two populations of wildebeest, a small population of resident wildebeests which are present all year round and thousands of wildebeests which migrate from the Serengeti to occupy the MMNR for a period of three to four months before they retreat [16,37]. The MMNR is an open conservation area, without fences and wildlife and livestock frequently graze in close proximity.

### 4.3. Wildebeest Sampling

Wildebeests were immobilized by darting using a combination of Etorphine hydrochloride (M99^®^) and Xylazine hydrochloride (Norvatis [PTY] Ltd, Gauteng, South Africa). The animals were reversed using a combination of Diprenophine and Atipamazole. Venous blood was drawn from the jugular vein and placed into 10 mL EDTA tubes, which was aliquoted into cryovials for storage in Liquid Nitrogen. A total of 32 blood samples were collected and preserved in ultra-cold freezer (−80 °C) at the Veterinary Services Laboratory of Kenya Wildlife Service (KWS), Nairobi. Twenty six samples were from migratory wildebeest whereas six were from resident wildebeest. Resident wildebeest and migratory wildebeest were identified because they occupy spatially distinct areas within the reserve.

### 4.4. DNA Extraction and Amplification

All frozen blood samples were thawed prior to DNA Extraction. DNA was extracted from 200 μL of blood, using the Qiagen DNeasy blood and tissue extraction kit (QIAGEN, Southern Cross Biotechnologies, Cape Town, South Africa) following the manufacturers protocol. DNA yields were determined with a Nanodrop^®^ ND-1000 Spectrophotometer (Nanodrop Technologies, Wilmington, DE, USA), and DNA was stored at −20 °C until subsequent analysis. We amplified the hypervariable V4 region of the 18S rRNA gene of the genera *Theileria* and *Babesia* from genomic DNA using a nested polymerase chain reaction. We used two sets of primers, one set for primary amplification and a second set for secondary amplification. Primary amplification was performed using primers; ILO-9029 (Forward), (5′-CGGTAATTCCAGCTCCAATAGCGT-3′) and ILO-9030 (Reverse) (5′-TTTCTCTCAAAGGTGCTGAAGGAGT-3′). The conditions for the primary amplification consisted of an initial denaturation step of 5 min at 95 °C, followed by 30 cycles of denaturation each at 95 °C for 30 s, annealing for 30 s at 53 °C, extension for 1 min at 72 °C and terminated by a final extension for 8.5 min at 72 °C. We carried out the secondary amplification by ILO-9029 (Forward), (5′-CGGTAATTCCAGCTCCAATAGCGT-3′) and ILO-7782 (Reverse) (5′-AACTGACGACCTCCAATCTCTAGTC-3′). The conditions for secondary amplification consisted of an initial denaturation step of 5 min at 95 °C, and 30 cycles of denaturation for 30 s at 95 °C, annealing for 30 s at 50 °C, extension for 1 min at 72 °C and terminated by a final extension for 8.5 min at 72 °C. We included extraction controls and PCR-product negative (water) in each PCR reaction as negative controls. We carried out both primary and secondary amplifications based on a final volume of 10 μL which consisted of 1.5 μL of genomic DNA, 0.1 μL of each primer, 5 μL of Thermo Scientific™ DreamTaq™ Green PCR Master Mix (2×) and 3.3 μL of water.

### 4.5. Detection Purification and Sequencing of PCR Products

The final PCR product (1 μL) was separated using gel electrophoresis on 1.5% agarose gel stained with GelRed™ Nucleic acid gel stain. The gel was visualized for positive amplification of the target region on a UV trans-illuminator, and photographed. One kb DNA Ladder was used to identify the approximate size of the molecule run on a gel. All PCR products that were positive upon visualization on agarose gel were purified and sequenced at Macrogen in Europe. The primers used to amplify the 18S rRNA during PCR was used to sequence PCR products in both the forward and reverse directions using Sanger method in an ABI 3730 Analyzer (Applied Biosystems, Carlsbad, California, USA).

### 4.6. Sequence and Phylogenetic Analyses

The chromatograms were visualized and edited using ChromasLite v 2.1.1. Primers were trimmed out using the software for Molecular Evolutionary Genetics Analysis (MEGA) v.6 [43]. The consensus nucleotide sequences were aligned using MUSCLE v. 3.8.31 [44] and visualized using SeaView v.4 [45]. Unique sequences, herein referred to as haplotypes were identified from aligned sequences using DnaSP v 5.10.01 [46]. Sequences that were Orthologous to our haplotypes were identified from GenBank [47] using the BLASTn algorithm [48]. We selected the closest sequence match to our haplotypes based on expectation value of greater than 1 × 10^−10^. All our haplotypes matched closely to *Theileria* species. In order to classify our haplotypes into species or clusters of species, at least three representative sequences of each known *Theileria* species from GenBank were obtained for comparison. Altogether, we had three sequences from wildebeests and 50 sequences from GenBank (NCBI) subjected to phylogenetic analysis.

Prior to phylogenetic analyses, multiple sequence alignment was performed using the program MUSCLE. MEGA v.6 was used to determine the model of sequence evolution as well as the rate heterogeneity of aligned sequences [43]. The phylogeny was inferred using the maximum likelihood method based on a Tamura-Nei (1993) model [49] with a gamma shape parameter to model the nucleotide substitution pattern and rate of evolution. Statistical support for internal branches of the trees or their reliability was evaluated by bootstrapping with 1000 iterations [50]. The resultant tree was viewed and edited in Dendroscope v.3 [51]. To test the robustness of the phylogenetic analyses, sequence clusters were also detected by the analysis of phylogenetic networks inferred from uncorrected p-distances with the phylogenetic split decomposition network implemented with Splits Tree v. 4.13.1 [52]. Phylogenetic network diagram produced from these analyses was used to validate the three new haplotypes. Lastly, we used DnaSP to investigate sequence divergence and polymorphism between our haplotypes and the GenBank references. Nucleotide divergence or the average number of nucleotide substitutions per site between haplotypes and was estimated using the Jukes and Cantor model. The section of the sequences with high polymorphism was detected using a sliding window in DnaSP and visualized using Jalview v 2.8.2 [53].

## 5. Conclusions and Recommendations

With the use of advanced molecular techniques we were able to identify *Theileria* species infecting wildebeest and their phylogenetic relationships with genetically characterized species in wild and domestic animals. This study contributes to the knowledge on the diversity of *Theileria* infecting wildlife and the new genotypes revealed by this study will be useful in PCR diagnostics for understanding cross transmission between domestic and wildlife species. The sharing of *Theileria* haplotypes between resident and migratory species suggests that migration of the wildebeests plays a central role in the spread of pathogens within the transboundary ecosystem and most likely posing transmission risks beyond conspecifics but also across heterospecifics, especially livestock (goats and sheep).

Further studies need to be conducted to determine the role of wildebeest in the transmission of multiple pathogens and whether the species found in wildebeest are also in livestock. The advent of molecular tools and increasing interest in wildlife pathogens is revealing numerous previously unknown species and variants of *Theileria* and *Babesia*, which is likely to alter several aspects of previously held notions, such as their host range, diversity, phylo-geography and ultimately classification.
